# Acute effects of air pollution on respiratory disease mortalities and outpatients in Southeastern China

**DOI:** 10.1038/s41598-018-19939-1

**Published:** 2018-02-22

**Authors:** Zhe Mo, Qiuli Fu, Lifang Zhang, Danni Lyu, Guangming Mao, Lizhi Wu, Peiwei Xu, Zhifang Wang, Xuejiao Pan, Zhijian Chen, Xiaofeng Wang, Xiaoming Lou

**Affiliations:** 1 Department of Environmental and Occupational Health, Zhejiang Provincial Center for Disease Control and Prevention, Binsheng Road 3399#, Hangzhou, 310051 Zhejiang Province China; 2 Eye Center of the 2nd Affiliated Hospital, School of Medicine, Zhejiang University, Zhejiang Provincial Key Lab of Ophthalmology, Jiefang Road 88#, Hangzhou, 310009 Zhejiang Province China

## Abstract

The objective of this study was to investigate the potential association between air pollutants and respiratory diseases (RDs). Generalized additive models were used to analyze the effect of air pollutants on mortalities or outpatient visits. The average concentrations of air pollutants in Hangzhou (HZ) were 1.6–2.8 times higher than those in Zhoushan (ZS), except for O_3_. In a single pollutant model, the increased concentrations of PM_2.5_, NO_2_, and SO_2_ were strongly associated with deaths caused by RD in HZ, while PM_2.5_ and O_3_ were associated with deaths caused by RD in ZS. All air pollutants (PM_2.5_, NO_2_, SO_2_, and O_3_) were strongly associated with outpatient visits for RD in both HZ and ZS. In multiple pollutant models, a significant association was only observed between PM_2.5_ and the mortality rate of RD patients in both HZ and in ZS. Moreover, strong associations between SO_2_, NO_2_, and outpatient visits for RD were observed in HZ and ZS. This study has provided evidence that both the mortality rates and outpatient visits for RD were significantly associated with air pollutants. Furthermore, the results showed that different air pollutant levels lead to regional differences between mortality rates and outpatient visits.

## Introduction

China is currently experiencing severe air pollution caused by increasing coal consumption, motor vehicle usage, and industrial dust, which are linked to rapid economic development^1^. The adverse impacts of air pollution on public health are enormous and have increased social concerns. An increasing number of studies have been conducted to investigate the associations between air pollution and certain diseases. Respiratory diseases have been found to have a close relationship with air pollution because the respiratory system is directly exposed to the external environment. Associations between air pollution and respiratory diseases have been observed in studies from many countries, including China^2–9^. Despite the increasing number of air quality studies conducted in China, air pollution epidemiology studies on the effects of PM_2.5_ and O_3_ in Chinese populations are still limited^10^.

Hangzhou (HZ) (between 119.982–120.388°E and 30.082–30.398°N) is one of the largest cities in the Yangtze River Delta (YRD) region, which is considered as one of the most rapidly developing regions in China. As a result of urbanization and industrialized processes, HZ has severe air pollution like many Chinese cities^11–13^. Several studies have been conducted on the detrimental effects of air pollution on residents’ health in HZ^2,14,15^ as part of nationwide investigations on China’s air pollution and the resulting health effects. However, most research on air pollution and respiratory diseases has been carried out in heavily polluted areas, so comparisons with other less polluted areas are lacking. Therefore, the city of Zhoushan (ZS) (between 121.932–122.257°E and 29.658–30.186 °N), an island city with the best air quality in the region^16^, was selected as a comparison region to quantify the effect of air pollution on residents in a less polluted area.

The objective of this study was to assess the effects of air pollutants such as PM_2.5_, SO_2_, NO_2_, and O_3_ on the mortality rates of respiratory disease (RD), including one subcategory of RD, chronic obstructive pulmonary disease (COPD), and on hospital outpatients with RD in two cities with high and low levels of air pollution.

## Results

During the study period, the average concentrations of the air pollutants PM_2.5_, SO_2_, NO_2_, and O_3_ in HZ and ZS were 60.12, 17.25, 49.54, and 92.17 μg/m^3^ and 31.28, 6.14, 22.93, and 93.32 μg/m^3^, respectively. The average concentrations of the air pollutants in HZ were 1.6–2.8 times higher than those in ZS (*P* < 0.01), except for O_3_ (*P* > 0.05) (Table 1). Table 2 shows that the correlations between the air pollutants and meteorological factors had a similar pattern for HZ and ZS. The average daily mortality counts for RD and COPD in HZ and ZS were 7.50 and 2.99 and 4.57 and 1.53, respectively. The daily outpatient counts in HZ and ZS of adult and child patients averaged 416.66 and 229.19 and 78.89 and 53.44, respectively (Table 3). Air pollutants, meteorological factors, and outcomes also showed a seasonal trend (Supplementary Figs S1, S2, and S3).

**Table 1:** Summary statistics of air pollutants and meteorological factors in both city from 2014–2015.

City	Variable	PM_2.5_ (μg/m^3^)	SO_2_ (μg/m^3^)	NO_2_ (μg/m^3^)	O_3_ (μg/m^3^)	Temperature (°C)	Relative humidity (%)	Pressure (hpa)
HZ	Mean	60.124	17.251	49.538	92.174	17.566	74.033	1011.510
	Standard Deviation	32.193	8.830	16.739	52.019	8.228	14.021	8.912
	Min	8.286	4.250	12.625	6.429	−0.100	27.000	989.300
	25^th^ Percentiles	37.250	10.625	37.250	50.625	10.000	65.000	1003.700
	Median	54.625	15.500	47.500	80.536	19.200	75.000	1011.550
	75^th^ Percentiles	75.714	21.375	59.000	133.429	24.200	85.000	1018.700
	Max	229.375	77.125	106.625	247.375	33.200	98.000	1031.100
ZS	Mean	31.286	6.138	22.930	92.322	17.104	80.452	1012.120
	Standard Deviation	21.766	4.170	13.085	32.092	7.488	11.528	8.550
	Min	3.000	2.000	2.000	2.000	0.725	39.000	980.500
	25^th^ Percentiles	17.000	3.000	14.000	72.000	10.600	74.000	1005.000
	Median	26.000	5.000	21.000	90.500	18.050	82.500	1011.950
	75^th^ Percentiles	39.000	8.000	29.000	111.000	23.475	89.000	1018.800
	Max	163.000	42.000	100.000	231.000	30.400	98.000	1030.300
	*t*	20.010	32.000	33.780	−0.070	1.120	−9.560	−1.330
	*P*	<0.01	<0.01	<0.01	0.948	0.262	<0.01	0.185

**Table 2:** Correlation coefficient between air pollutants and meteorological factors in both cities from 2014–2015.

City	Variable	PM_2.5_ (μg/m^3^)	SO_2_ (μg/m^3^)	NO_2_ (μg/m^3^)	O_3_ (μg/m^3^)	Temperature (°C)	Relative humidity (%)	Pressure (hpa)
HZ	PM_2.5_ (μg/m^3^)	1.000						
	SO_2_ (μg/m^3^)	0.635^**^	1.000					
	NO_2_ (μg/m^3^)	0.670^**^	0.652^**^	1.000				
	O_3_ (μg/m^3^)	−0.006	−0.048	−0.319^**^	1.000			
	Temperature (°C)	−0.323^**^	−0.461^**^	−0.489^**^	0.638^**^	1.000		
	Relative humidity (%)	−0.253^**^	−0.533^**^	−0.132^**^	−0.415^**^	0.120^**^	1.000	
	Pressure (hpa)	0.339^**^	0.567^**^	0.487^**^	−0.474^**^	−0.891^**^	−0.269^**^	1.000
ZS	PM_2.5_ (μg/m^3^)	1.000						
	SO_2_ (μg/m^3^)	0.437^**^	1.000					
	NO_2_ (μg/m^3^)	0.569^**^	0.444^**^	1.000				
	O_3_ (μg/m^3^)	0.188^**^	0.130^**^	−0.098^**^	1.000			
	Temperature (°C)	−0.284^**^	−0.069	−0.235^**^	0.211^**^	1.000		
	Relative humidity (%)	−0.321^**^	−0.437^**^	−0.147^**^	−0.183^**^	0.362^**^	1.000	
	Pressure (hpa)	0.238^**^	0.151^**^	0.190^**^	−0.185^**^	−0.863^**^	−0.496^**^	1.000

^*^P < 0.05, ^**^P < 0.01.

**Table 3:** Summary statistics of respiratory mortalities and outpatients in both cities from 2014–2015.

City	Variable	N^a^	Mean	S. D^b^	Min	P_25_^c^	Median	P_75_^d^	Max
HZ	**Mortality counts**								
	RD	5477	7.503	3.775	0	5	7	10	29
	<65 years	231	0.316	0.615	0	0	0	1	5
	≥65 years	5246	7.186	3.585	0	5	7	9	24
	Male	3057	4.188	2.479	0	2	4	6	16
	Female	2420	3.315	2.150	0	2	3	5	13
	COPD	3338	4.573	2.663	0	3	4	6	18
	<65 years	72	0.099	0.312	0	0	0	0	2
	≥65 years	3266	4.474	2.609	0	3	4	6	17
	Male	1938	2.655	1.849	0	1	2	4	11
	Female	1400	1.918	1.519	0	1	2	3	10
	**Outpatient counts** ^e^								
	RD in adults	146665	416.662	116.487	134	344	416	465	866
	RD in children	80675	229.190	66.749	59	186	223	271	395
ZS	**Mortality counts**								
	RD	2185	2.993	2.055	0	1	3	4	10
	<65 years	118	0.162	0.414	0	0	0	0	3
	≥65 years	2067	2.832	1.959	0	1	3	4	10
	Male	1044	1.430	1.296	0	0	1	2	6
	Female	1141	1.563	1.384	0	0	1	2	6
	COPD	1117	1.530	1.495	0	0	1	2	10
	<65 years	74	0.101	0.332	0	0	0	0	3
	≥65 years	1043	1.429	1.418	0	0	1	2	10
	Male	560	0.767	0.952	0	0	1	1	6
	Female	557	0.763	0.985	0	0	0	1	6
	**Outpatient counts** ^e^								
	RD in adults	28717	78.893	28.025	21	61	78	95	193
	RD in children	19453	53.442	24.828	10	38	48	61	140

^a^Observations; ^b^Standard Deviation; ^c^25^th^ Percentiles; ^d^75^th^ Percentiles; ^e^The data was collected in 2014.

The associations between mortalities or outpatient visits and air pollutants were adjusted for potential confounding factors in single-pollutant models as presented in Table 4 and Supplementary Tables S1 and S2. An increase of 10 μg/m^3^ of air pollutants was significantly associated with the following: The ER of mortality of RD increased by 0.99 (95% CI: 0.03–1.95) for PM_2.5_ in HZ and by 2.09 (95% CI: 0.03–4.18) for PM_2.5_ in ZS. The ER of mortality of COPD increased by 1.60 (95% CI: 0.46–2.76), 6.33 (95% CI: 1.72–11.15), and 3.97 (95% CI: 1.58–6.41) for PM_2.5_, SO_2_, and NO_2_, respectively, in HZ, whereas no associations were identified in ZS. Outpatient visits of adults with RD increased by 0.67 (95% CI: 0.50–0.84), 3.50 (95% CI: 2.92–4.09), 2.10 (95% CI: 1.76–2.44), and −0.65 (95% CI: −0.83–0.47) for PM_2.5_, SO_2_, NO_2_, and O_3_, respectively, in HZ and also increased by 0.83 (95% CI: 0.23–1.43), 5.81 (95% CI: 3.12–8.58), 3.47 (95% CI: 2.41–4.54), and 0.61 (95% CI: 0.15–1.07) for PM_2.5_, SO_2_, NO_2_, and O_3_, respectively, in ZS. Outpatient visits of children with RD increased by 1.47 (95% CI: 1.22–1.71), 5.70 (95% CI: 4.92–6.49), 4.04 (95% CI: 3.57–4.51), and 0.21 (95% CI: 0.03–0.40) for PM_2.5_, SO_2_, NO_2_, and O_3_, respectively, in HZ and also increased by 1.78 (95% CI: 1.05–2.51), 10.89 (95% CI: 7.38–14.52), 8.02 (95% CI: 6.67–9.38), and 0.84 (95% CI: 0.29–1.40) for PM_2.5_, SO_2_, NO_2_, and O_3_, respectively, in ZS. The best lag day model that was incorporated into the single-pollutant models is shown in Table 4.

**Table 4:** Excess Risk of respiratory mortalities and outpatients per 10 μg/m^3^ increase of air pollutants in both cities with best lag: single-pollutant model.

Pollutant	Class	Variable	HZ		ZS	
			Lag	ER(95% CI)^a^	Lag	ER(95% CI)^a^
PM_2.5_	Mortality counts	RD	0	0.985(0.034–1.945)^*^	1	2.085(0.032–4.180)^*^
		Male	0	1.380(0.109–2.668)^*^	6	2.616(−0.290–5.607)
		Female	2	1.151(−0.168–2.487)	1	3.226(0.418–6.112)^*^
		COPD	1	1.601(0.456–2.76)^**^	6	2.377(−0.504–5.341)
		Male	1	1.666(0.169–3.185)^*^	6	4.664(0.668–8.819)^*^
		Female	1	1.537(−0.235–3.340)	2	3.133(−0.984–7.421)
	Outpatient counts	Adults^b^	4	0.671(0.500–0.842)^**^	5	0.830(0.230–1.433)^**^
		Children^c^	4	1.465(1.221–1.709)^**^	4	1.779(1.052–2.512)^**^
SO_2_	Mortality counts	RD	6	2.829(−0.703–6.487)	6	−7.156(−16.759–3.554)
		Male	6	4.471(−0.279–9.447)	3	3.823(−10.360–20.250)
		Female	0	−5.268(−10.678–0.469)	6	−12.191(−24.878–2.639)
		COPD	6	6.329(1.717–11.151)^**^	4	−5.842(−19.668–10.364)
		Male	6	4.921(−0.996–11.191)	3	15.035(−5.454–39.965)
		Female	5	8.797(1.639–16.46)^*^	6	−17.339(−35.525–5.977)
	Outpatient counts	Adults^b^	1	3.500(2.919–4.085)^**^	3	5.814(3.123–8.576)^**^
		Children^c^	2	5.704(4.923–6.491)^**^	1	10.894(7.379–14.524)^**^
NO_2_	Mortality counts	RD	4	1.478(−0.355–3.345)	5	−2.579(−5.810–0.764)
		Male	1	2.173(−0.397–4.809)	5	−2.270(−7.000–2.700)
		Female	5	2.536(−0.294–5.445)	5	−2.805(−7.176–1.772)
		COPD	4	3.969(1.583–6.412)^**^	0	3.547(−1.128–8.443)
		Male	4	3.269(0.183–6.450)^*^	5	−3.525(−9.854–3.247)
		Female	5	5.602(1.804–9.542)^**^	0	5.996(−0.602–13.032)
	Outpatient counts	Adults^b^	5	2.099(1.762–2.438)^**^	5	3.468(2.409–4.539)^**^
		Children^c^	2	4.042(3.573–4.514)^**^	4	8.018(6.672–9.381)^**^
O_3_	Mortality counts	RD	0	−0.529(−1.501–0.454)	2	1.928(0.302–3.580)^*^
		Male	3	−0.689(−1.647–0.279)	3	2.442(0.102–4.836)^*^
		Female	3	0.758(−0.330–1.857)	1	3.504(1.195–5.865)^**^
		COPD	1	−0.560(−1.550–0.440)	2	1.899(−0.366–4.217)
		Male	0	−0.939(−2.570–0.720)	3	3.138(−0.031–6.408)
		Female	1	−1.052(−2.564–0.485)	2	2.623(−0.594–5.945)
	Outpatient counts	Adults^b^	1	−0.653(−0.831–0.474)^**^	1	0.608(0.153–1.066)^**^
		Children^c^	2	0.211(0.025–0.397)^*^	2	0.842(0.292–1.395)^**^

^*^P < 0.05, ^**^P < 0.01 (Excess Risk is adjusted for temperature, relative humidity and atmospheric pressure, day of week, time trend and seasonality for mortality and hospital data, and public holiday only for hospital data); ^a^Excess Risk (95% confidence interval); ^b^RD in adults; ^c^RD in children.

The effects of air pollutants on mortalities and outpatients for the multiple-pollutant model are presented in Table 5. In this model, PM_2.5_, SO_2_, NO_2_, and O_3_ were included, and the lag days were selected based on the results of the single-pollutant model (Table 3). After adjusting for other air pollutants, PM_2.5_ in HZ was significantly associated with the mortality rates of RD and COPD, particularly with the mortality rates of COPD in both males and females. The effect was slightly enhanced compared to the results of the corresponding single-pollutant model. In HZ, NO_2_ was significantly associated with the mortality rates of COPD; meanwhile, in ZS, O_3_ was significantly associated with the mortality rates of RD, especially RD mortalities in females. In both cases, the effects were slightly lower compared to the corresponding single-pollutant models. For outpatient visits of individuals with RD, SO_2_ and NO_2_ were significantly associated with outpatient visits of adults and children in both HZ and ZS; only PM_2.5_ was significantly associated with outpatient visits of children in HZ. The effects were slightly lower in comparison to the single-pollutant models. In HZ, O_3_ was significantly associated with adult outpatient visits; this association was negative in the single-pollutant model but positive in the multiple-pollutant model.

**Table 5:** Excess Risk of respiratory mortalities and outpatients per 10 μg/m^3^ increase of air pollutants in both cities: multiple-pollutant model.

Pollutant	Class	Variable	ER(95% CI) in HZ^a^	ER(95% CI) in ZS^a^
PM_2.5_	Mortality counts	RD	1.290(0.294–2.295)^*^	1.969(−0.183–4.167)
		Male	1.344(−0.068–2.777)	2.744(−0.330–5.913)
		Female	0.854(−0.492–2.218)	2.598(−0.461–5.750)
		COPD	1.737(0.521–2.967)^**^	2.669(−0.304–5.731)
		Male	1.788(0.242–3.359)^*^	4.795(0.510–9.262)^*^
		Female	1.931(0.076–3.821)^*^	2.111(−2.462–6.899)
	Outpatient counts	Adults^b^	0.132(−0.059–0.323)	−0.165(−0.892–0.568)
		Children^c^	0.465(0.210–0.720)^**^	0.261(−0.534–1.062)
SO_2_	Mortality counts	RD	1.660(−2.017–5.475)	−6.473(−16.716–5.030)
		Male	3.621(−1.195–8.671)	2.839(−12.338–20.645)
		Female	−5.286(−11.196–1.018)	−12.616(−26.064–3.277)
		COPD	4.101(−0.651–9.082)	−11.051(−24.587–4.914)
		Male	2.967(−3.150–9.469)	16.951(−5.303–44.435)
		Female	3.469(−4.983–12.674)	−20.743(−38.655–2.399)
	Outpatient counts	Adults^b^	1.882(1.214–2.554)^**^	2.813(0.075–5.625)^*^
		Children^c^	2.150(1.239–3.069)^**^	4.482(0.844–8.252)^*^
NO_2_	Mortality counts	RD	1.201(−0.745–3.186)	−1.579(−5.057–2.026)
		Male	0.966(−1.870–3.884)	−3.247(−8.204–1.978)
		Female	2.620(−0.251–5.574)	−1.086(−5.805–3.870)
		COPD	2.890(0.369–5.474)	3.869(−1.024–9.004)
		Male	2.381(−0.900–5.772)	−6.164(−12.719–0.884)
		Female	4.153(−0.476–8.998)	6.787(−0.135–14.188)
	Outpatient counts	Adults^b^	1.469(1.093–1.846)^**^	1.324(0.002–2.664)^*^
		Children^c^	2.098(1.581–2.617)^**^	2.032(0.679–3.404)^**^
O_3_	Mortality counts	RD	−0.700(−1.699–0.309)	1.879(0.230–3.554)^*^
		Male	−0.603(−1.567–0.371)	2.090(−0.308–4.545)
		Female	0.589(−0.516–1.707)	3.010(0.557–5.524)^*^
		COPD	−0.806(−1.832–0.231)	2.175(−0.120–4.523)
		Male	−0.975(−2.637–0.716)	2.325(−0.878–5.632)
		Female	−1.201(−2.773–0.396)	2.351(−1.084–5.906)
	Outpatient counts	Adults^b^	0.222(0.062–0.383)^**^	0.348(−0.117–0.814)
		Children^c^	−0.198(−0.396–0.000)	−0.249(−0.814–0.320)

^*^P < 0.05,^**^P < 0.01 (Excess Risk is adjusted for temperature, relative humidity and atmospheric pressure, day of week, time trend and other pollutants for mortality and hospital data, and public holiday only for hospital data); ^a^Excess Risk (95% confidence interval); ^b^RD in adults; ^c^RD in children.

With respect to gender (Table 5 and Supplementary Table S3), the ER values for the mortality rates of COPD as a result of PM_2.5_ concentrations in the atmosphere were higher for females than males in HZ. These patterns were also observed in the mortality rates of RD stemming from O_3_ in ZS; however, an opposite trend was identified for the mortality rates of COPD stemming from PM_2.5_ in ZS. Seasonal differences in air pollutants were also observed between HZ and ZS and likely affect the rates of RD and COPD (Supplementary Table S4).

## Discussion

During this study, the different impacts of air pollutants on respiratory mortality rates and outpatient visits were evaluated and compared between HZ and ZS, which have distinct air pollution levels. In particular, SO_2_ and NO_2_ were strongly associated with respiratory mortality rates and outpatient visits in HZ, and the concentrations of SO_2_ and NO_2_ were about 2.81 and 2.16 times higher, respectively, in HZ than in ZS. Moreover, O_3_ appeared to play a more important role in respiratory mortality rates and outpatient visits in areas with a lower level of air pollution than in areas with a higher level of air pollution.

Because of rapid economic development, HZ has become one of the most heavily air-polluted cities in China. The results of the multiple-pollutant model in this study showed that RD increased 1.29% per 10 μg/m^3^ increase in PM_2.5_, which is slightly higher than other cities in eastern China (0.95–0.99%) with similar average concentrations of PM_2.5_^8,17,18^. This finding is in line with the results of a study conducted across all WHO regions (1.51%)^19^ but reflects lower risk than the results of another study conducted in several cities in the US, Western Europe, and South Korea with lower average concentrations of PM_2.5_ (1.68–3.90%)^20–24^.

Previous studies on exposure to SO_2_ and RD have not reached a coherent conclusion. However, a positive association between SO_2_ exposure and RD has been reported by several groups. A cross-case study showed that an increased number of asthma episodes was associated with elevated exposure to SO_2_ emitted from refinery factories^25^. Another study indicated that children living near a petrochemical site had a higher prevalence of respiratory hospitalizations and symptoms^26^. In contrast, no associations or only moderate associations between SO_2_ exposure and RDs have been reported in other studies. A cross-sectional study in Taiwan showed that the prevalence rate of sinusitis, wheezing, asthma, allergic rhinitis, bronchitis, and pneumonia were not significantly higher in children exposed to a high level of SO_2_^27^. Similarly, another study showed no association between SO_2_ concentrations and emergency room visits^28,29^. One possible explanation for these inconsistent results may involve the regional differences in air pollution levels and the diverse compositions of air pollutants. Therefore, to reduce the influence of regional differences in the association between SO_2_ exposure and RD, the present study investigated two cities in the same province: HZ, which has severe air pollution, and ZS, which has mild air pollution. A significant association between COPD mortality rates and SO_2_ was only observed in HZ in the single-pollutant model, whereas no positive correlations were found in ZS. However, outpatient visits for RD were strongly associated with SO_2_ exposure in HZ and ZS for both adults and children; this association was stronger in ZS than in HZ, even though the concentration of SO_2_ was 2.81 times higher in HZ than in ZS. These results indicate that the association between SO_2_ and outpatient visits for RD may also depend on individual sensitivity, which could partially explain the inconsistent results of previous studies with respect to the influence of SO_2_ on RDs.

The adverse effects of PM_2.5_ on human health have attracted increasing public attention. Studies conducted in Xi’an and Guangzhou, two industrialized cities in China, reported that an increase in mortality rates was significantly associated with an increase in PM_2.5_ concentrations^18,30^, which would also suggest a significant impact on RD. In the present study, the mortality rates of RD and COPD in males were associated with a 10 μg/m^3^ increase in the concentration of PM_2.5_ in both HZ and ZS, which is consistent with the results of previous studies; however, the association was stronger in ZS, which had a lower concentration of PM_2.5_, than in HZ, which had a higher concentration of PM_2.5_. This may be explained by differences in sample sizes, climates, lifestyles, etc.

Studies in the Pearl River Delta region of Southern China and in Shanghai reported that O_3_ exposure was also associated with mortality risks^31,32^. In the present study, the mortality rates of RD were significantly associated with O_3_ in both males and females in ZS, while no association between the mortality rates of RD and O_3_ was observed in HZ. It seems that the effects of O_3_ on mortality rates could be attenuated by other air pollutants, which is partially supported by the correlation coefficients of the analyzed air pollutants in the present study.

In addition, NO_2_, another principal air pollutant in China, was reported to be linked to deaths from RD^33^. The present results showed that NO_2_ was associated with deaths from COPD in HZ but not in ZS; however, there was a significant association between outpatient visits for RD resulting from NO_2_ pollution in both HZ and ZS. These results are consistent with the findings for SO_2_, indicating that some air pollutants may need to reach a specific threshold to cause adverse effects on human health.

The present study has several strengths. First, the investigations were conducted in two cities with different levels of air pollution in the same province. Second, the number of outpatient visits as well as mortality rates was used to analyze the effects of air pollutant levels on human health. Third, four high-ranking air pollutants were selected to investigate the relationship between air pollution and RD.

The limitations of the analysis should also be noted. One limitation of our study was that although the hospitals included in this study were the pulmonary hospital in HZ and the largest hospital in ZS, some patients may have visited other hospitals. Therefore, additional hospitals should be included in future studies. Another limitation was that RD outpatients were not divided into more detailed subgroups including acute upper respiratory tract infections, COPD, pneumonia, etc., so the responses of different types of RD to air pollutants could not be investigated in our study. The effects of air pollutants on subgroups of RD should be further investigated in future studies.

## Conclusions

In summary, a significant association between air pollutants and RD was found both in terms of mortality rates and outpatient visits. Also, the different air pollutant levels in HZ and ZS lead to regional differences in mortality rates and outpatient visits. In line with previous studies, these results suggest that the government should accelerate the implementation of environmental protection policies to improve the quality of life of its citizens.

## Materials and Methods

### Air pollution and meteorology data

**Figure 1:**
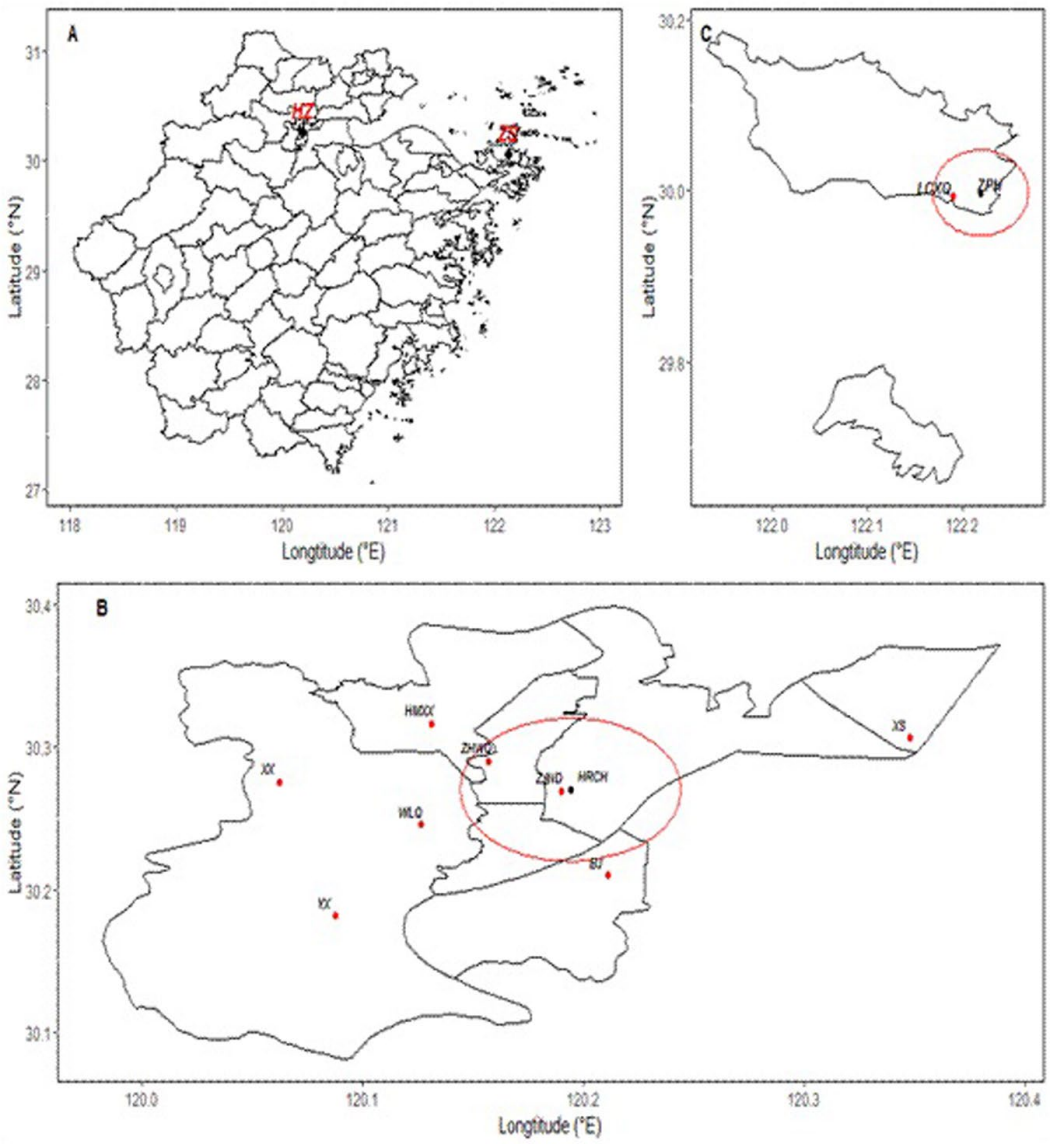
Locations of environmental monitoring stations and hospitals in HZ and ZS of the Zhejiang Province, China: (**A**) Map of the Zhejiang Province; (**B**) Map of urban HZ: the red points are the locations of stations; the black point is the location of the hospital; the labels on the map are abbreviations according to the first letter of the name; the red circle on the map is the 10-km range of Hangzhou Red Cross Hospital (HRCH). The map shows that the location of Zhaohuiwuqu (ZHWQ) and Zhejiangnongda (ZJND) both fall within the 10-km range of HRCH. (**C**) Map of urban ZS: the red points are the locations of stations; the black point is the location of the hospital; the labels on the map are abbreviations according to the first letter of the name; the red circle on the map is the 10 km range of Zhoushan People’s Hospital (ZPH). The map shows that the location of Linchengxinqu (LCXQ) falls within the 10-km range of ZPH. The figure was generated by R 64 3.3.1 with Package *ggplot 2* version 2.2.1 (https://cran.r-project.org/web/packages/ggplot2/index.html) (H.Wickham.ggplot2: Elegant Graphics for Data Analysis. Springer-Verlag New York, 2009).

Concentrations of PM_2.5_, SO_2_, NO_2_, and O_3_ were collected between 1 January, 2014 and 31 December, 2015, from eight environmental monitoring stations in urban areas of HZ and from one environmental monitoring station in an urban area of ZS. Daily mean temperature, relative humidity, and atmospheric pressure measurements were provided by the Zhejiang Meteorological Administration. Twenty-four-h means were used for all air pollutants except O_3_, which is conventionally measured at a maximum interval of 8 h. The locations of the stations are shown in Fig. 1 and Supplementary Table S5 (HZ: Hemuxiaoxue, Xixi, Yunxi, Zhejiangnongda, Binjiang, Xiasha, Wolongqiao, and Zhaohuiwuqu; ZS: Linchengxinqu).

### Mortality data

The mortality records for RD in HZ and ZS during the study period were obtained from the local mortality register of the Zhejiang Provincial Center for Disease Prevention and Control. These records were encoded using the 10^th^ revision of the international classification of diseases and related health problems (ICD-10)^34^ and included the dates of mortality, ages, genders, and addresses of deceased patients.

### Hospital outpatient data

Outpatient data for RD were obtained between 1 January, 2014 and 31 December, 2014, in one tertiary hospital located in HZ and another located in ZS. The data obtained from these hospitals are geographically representative because patients in these hospitals generally reside in the corresponding local areas. These data were also encoded using ICD-10. Patients who visited either the same doctor or multiple respiratory doctors more than once within 15 days were excluded from the data. The locations of the hospitals are provided in Fig. 1 and Supplementary Table S5 (Hangzhou Red Cross Hospital; Zhoushan People’s Hospital).

### Data analysis

The diseases were categorized as RD (ICD-10 codes J00-99) or COPD (ICD-10 codes J40-44). The mortality data were divided into two gender groups, male and female, and two age groups, <65 years (non-elderly) and ≥65 years (elderly), as most deaths occurred in the elderly. Because of the relatively low daily mortality rate in the <65 years age group, the effect of air pollutants on mortality rates for this age group was not analyzed. The outpatient data were divided into two groups, RD in adults (age > 18) and RD in children (age ≤ 18). Patients younger than 18 years often visit pediatric clinics in China, so data from both adult and pediatric clinics were used. The whole year was divided into two seasons, a warm season (April to September) and a cold season (January to March and October to December), according to the seasonal characteristics of HZ and ZS. Only the mortality records that included current residential addresses within the range of the above mentioned stations were analyzed. These data from records were examined along with the mean concentrations of air pollutants from all stations according to city and date. The distance from each hospital to the stations was determined using the SoDA package in R. The data collected from stations within 10 km^35^ of each hospital were averaged. The Zhaohuiwuqu and Zhejiangnongda stations were paired with the hospital in HZ; the Linchengxinqu station was paired with the hospital in ZS (Fig. 1).

For all analyses, the level of significance was set at *P* < 0.05. The descriptive statistics and correlations were calculated for mortalities, outpatient visits, air pollutants, and meteorological factors using the SAS 9.2 software (Cary, NC, USA). The *t* test was used to compare the continuous variables, and the Spearman correlation was used to evaluate the correlations among the air pollutants and the meteorological factors.

The associations between the mortalities of outpatients and air pollutants were adjusted for meteorological factors, days of the week, public holidays, time trends, and seasonality and were estimated using generalized additive models (GAM)^36^ in the MGCV packages in R vision 3.3.1. The weather conditions, including daily mean temperature, relative humidity, and atmospheric pressure, were controlled using natural spline smoothing functions. The adjustments for time trends and seasonality were made using natural spline smoothing functions of time. The degrees of freedom (*df*) for the functions were determined via generalized cross validation (GCV)^37^. In addition, dummy variables were used for days of the week and public holidays to control for potential confounding factors. To control for any lag effect, the concentrations of air pollutants on the current day (lag0) and the previous six days (lag1–6) were incorporated into the model. The best time lag model was selected according to the minimum *P* values; only single lag models were considered. Using residual plots and partial autocorrelation function (PACF) plots in the TSA packages in R vision 3.3.1, the residual models were examined to determine the presence of autocorrelations^2^. All results were presented as excess risk (ER) of outpatient mortality per 10 μg/m^3^ increase of each air pollutant in comparison to the baseline at a 95% confidence interval (CI). The resulting model was as follows:

$$\mathrm{Log}[{\rm{E}}({{\rm{Y}}}_{{\rm{t}}})]=\alpha +\beta {Z}_{{\rm{t}}-{\rm{i}}}+{\rm{ns}}\,(time,\,df)+{\rm{ns}}\,({X}_{t},\,df)+DOW+holiday$$

where Y_t_ is the number of daily respiratory mortalities or outpatients at day t; E(Y_t_) is the expectation of the Poisson distribution of Y_t_; *α* is the intercept; Z_t−i_ is the concentration of air pollutants in lag(i) day, i = 0 to 6; *β* is the regression coefficient; ns (*time*, *df*) refers to the natural spline smoothing functions of the day of study (*df* = 2 × 2); ns (*X*_*t*_, *df*) refers to the natural spline smoothing functions of meteorological factors, such as daily mean temperature (*df* = 3), relative humidity (*df* = 3), and atmospheric pressure (*df* = 3); *DOW* represents the dummy variables for the day of the week; and holiday is the dummy variable for public holidays, which was only used in the model for outpatient visits. The significant differences between the values of the ER of the group variables (e.g., male and female) were determined by calculating the 95% CI as follows:

$$(\hat{Q}{\rm{1}}-\hat{Q}{\rm{2}})\pm 1.96\sqrt{S\hat{E}{{\rm{1}}}^{2}+S\hat{E}{2}^{{\rm{2}}}}$$

where $\hat{Q}1$ and $\hat{Q}{\rm{2}}$ are the values of ER for the two groups, and $S\hat{E}1$ and $S\hat{E}2$ are their respective standard errors^38^.

## Electronic supplementary material

Supplemental data
