# Supplementary material for: Acute effects of air pollution on respiratory disease mortalities and outpatients in Southeastern China
Source: Sci Rep. 2018 Feb 22;8:3461. doi: 10.1038/s41598-018-19939-1 (PMC5823896; doi:10.1038/s41598-018-19939-1)
Supplement: Supplementary file 1 — Supplemental data [file 41598_2018_19939_MOESM1_ESM.doc]

**Acute effects of air pollution on respiratory disease mortalities and outpatients in Southeastern China**

Zhe Mo^1#^, Qiuli Fu^2#^, Lifang Zhang^2^, Danni Lyu^2^, Guangming Mao^1^, Lizhi Wu^1^, Peiwei Xu^1^, Zhifang Wang^1^, Xuejiao Pan^1^, Zhijian Chen^1*^, Xiaofeng Wang^1*^, Xiaoming Lou^1^*

**Supplemental figures**

**
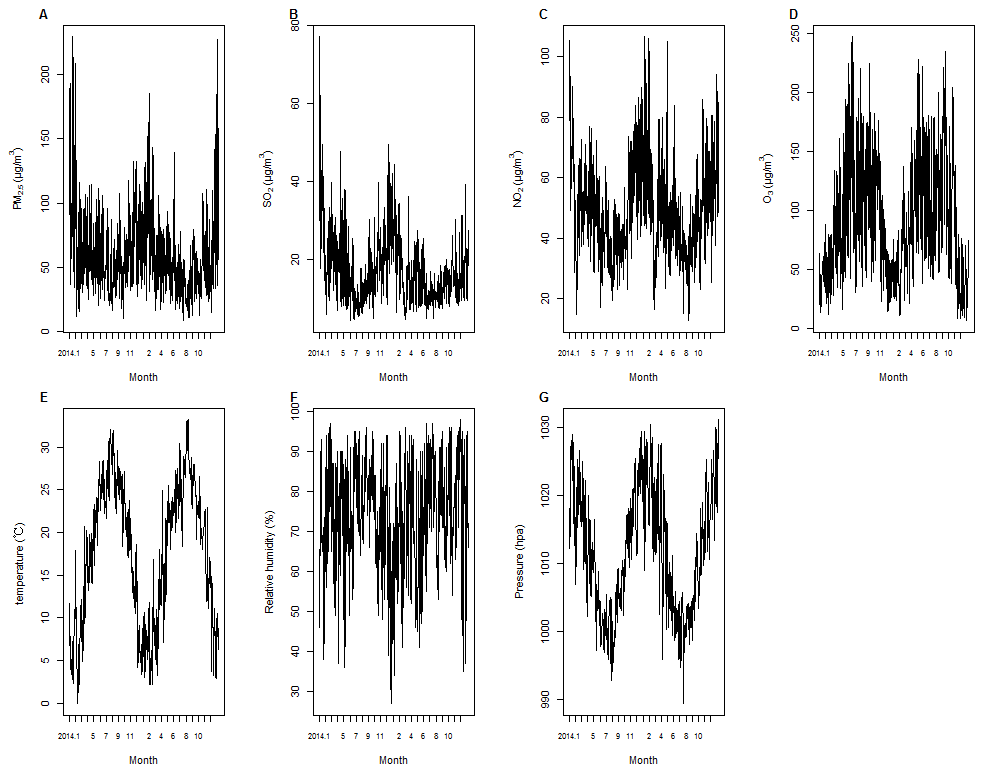
**

**Supplementary Figure S1.** Time series plots of daily concentrations of PM_2.5_, SO_2_, NO_2_, O_3_, temperature, relative humidity and atmospheric pressure in HZ, 2014-2015

**
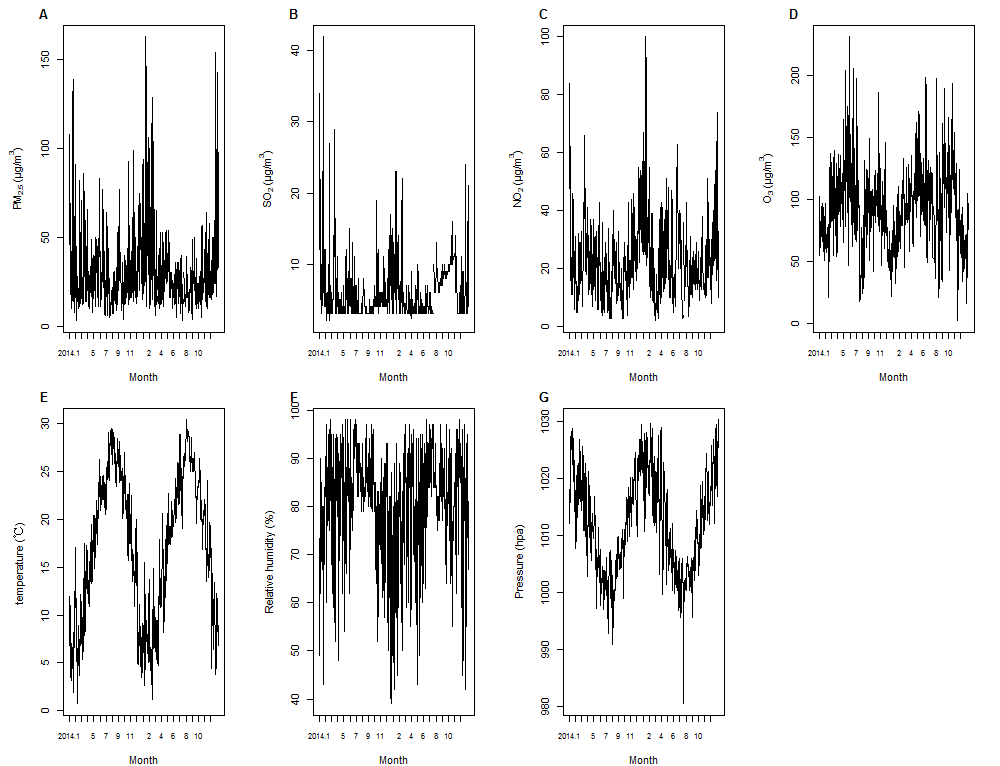
**

**Supplementary Figure S2.** Time series plots of daily concentrations of PM_2.5_, SO_2_, NO_2_, O_3_, temperature, relative humidity and atmospheric pressure in ZS, 2014-2015

**
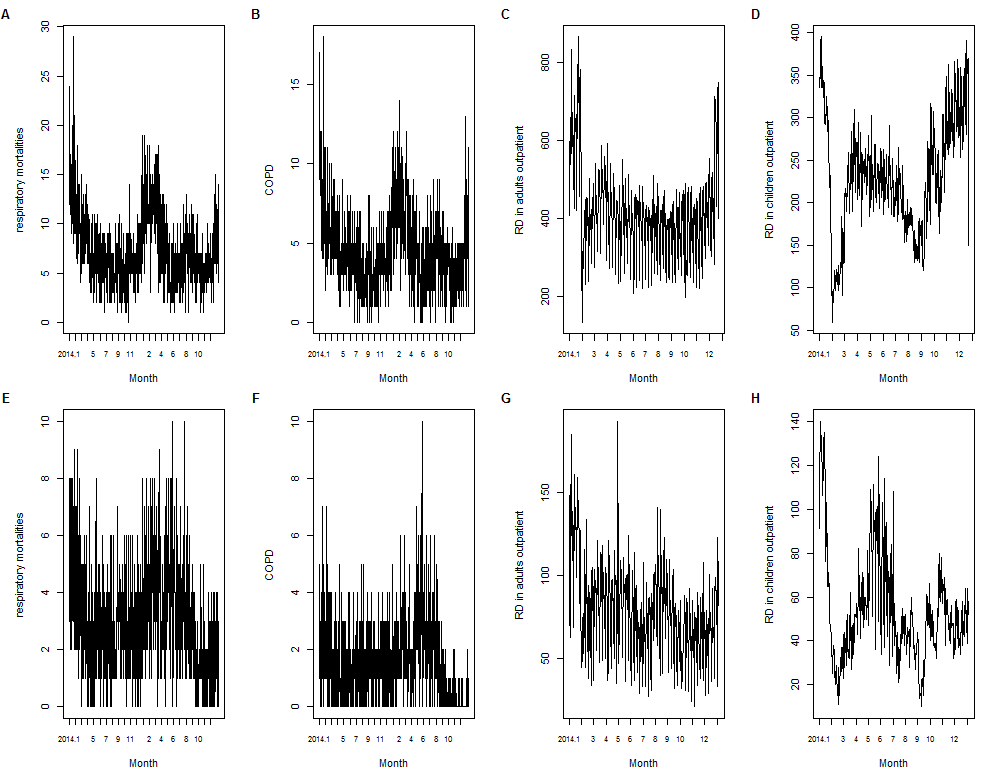
**

**Supplementary Figure S3.** Time series plots of daily mortality for respiratory disease in HZ and ZS, 2014-2015, outpatient for respiratory disease in HZ and ZS, 2014. A-D for HZ, E-H for ZS.

**Supplemental Tables**

**Supplementary Table S1** Excess Risk (95% confidence interval) of respiratory mortalities and outpatients per 10 μg/m^3^ increase of air pollutants for different lag days in HZ: single-pollutant model

窗体顶端

窗体底端

| Variable | Lag | PM_2.5_ | SO_2_ | NO_2_ | O_3_ |
| --- | --- | --- | --- | --- | --- |
| Mortality counts |  |  |  |  |  |
| RD | 0 | 0.985(0.034-1.945)^*^ | -2.678(-8.338-3.331) | 0.27(-1.56-2.134) | -0.529(-1.501-0.454) |
|  | 1 | 0.8(-0.098-1.706) | 0.436(-2.974-3.966) | 1.403(-0.504-3.345) | -0.402(-1.176-0.377) |
|  | 2 | 0.76(-0.126-1.653) | 0.382(-3.001-3.884) | 1.03(-0.848-2.944) | -0.296(-1.017-0.431) |
|  | 3 | 0.502(-0.369-1.38) | 1.67(-1.74-5.197) | 0.499(-1.34-2.373) | -0.052(-0.772-0.673) |
|  | 4 | 0.37(-0.503-1.25) | 1.866(-1.553-5.403) | 1.478(-0.355-3.345) | 0.236(-0.49-0.967) |
|  | 5 | 0.138(-0.736-1.02) | 0.052(-3.387-3.613) | 1.176(-0.688-3.075) | 0.263(-0.465-0.996) |
|  | 6 | 0.381(-0.495-1.265) | 2.829(-0.703-6.487) | 0.921(-0.97-2.847) | -0.038(-0.769-0.698) |
| Male | 0 | 1.38(0.109-2.668) ^*^ | -4.176(-9.076-0.989) | 1.249(-1.22-3.78) | -0.721(-2.021-0.596) |
|  | 1 | 0.993(-0.207-2.207) | 2.018(-2.569-6.821) | 2.173(-0.397-4.809) | -0.211(-1.247-0.836) |
|  | 2 | 0.451(-0.741-1.656) | 0.521(-4.001-5.256) | 1.238(-1.275-3.815) | -0.508(-1.471-0.465) |
|  | 3 | 0.342(-0.823-1.521) | 1.231(-3.295-5.968) | -0.031(-2.47-2.469) | -0.689(-1.647-0.279) |
|  | 4 | 0.223(-0.94-1.399) | 1.149(-3.37-5.879) | 0.792(-1.635-3.278) | 0.104(-0.867-1.085) |
|  | 5 | -0.052(-1.22-1.131) | -1.183(-5.705-3.555) | 0.119(-2.342-2.641) | 0.63(-0.346-1.616) |
|  | 6 | 0.916(-0.249-2.095) | 4.471(-0.279-9.447) | 1.773(-0.76-4.37) | 0.11(-0.868-1.097) |
| Female | 0 | 0.495(-0.931-1.942) | -5.268(-10.678-0.469) | -0.949(-3.65-1.829) | -0.283(-1.744-1.199) |
|  | 1 | 0.565(-0.784-1.933) | -1.522(-6.542-3.767) | 0.439(-2.378-3.339) | -0.637(-1.796-0.535) |
|  | 2 | 1.151(-0.168-2.487) | 0.257(-4.766-5.546) | 0.775(-2.029-3.659) | -0.024(-1.108-1.071) |
|  | 3 | 0.709(-0.597-2.032) | 2.195(-2.907-7.566) | 1.181(-1.597-4.037) | 0.758(-0.33-1.857) |
|  | 4 | 0.57(-0.745-1.903) | 2.769(-2.375-8.185) | 2.354(-0.418-5.205) | 0.408(-0.683-1.511) |
|  | 5 | 0.378(-0.933-1.706) | 1.665(-3.545-7.157) | 2.536(-0.294-5.445) | -0.202(-1.29-0.898) |
|  | 6 | -0.322(-1.644-1.017) | 0.739(-4.457-6.218) | -0.155(-2.97-2.742) | -0.226(-1.323-0.884) |
| COPD | 0 | 0.951(-0.269-2.187) | -4.411(-9.073-0.49) | 0.757(-1.591-3.16) | -0.582(-1.827-0.679) |
|  | 1 | 1.601(0.456-2.76)^**^ | 3.676(-0.764-8.313) | 3.365(0.878-5.914) ^**^ | -0.56(-1.55-0.44) |
|  | 2 | 0.957(-0.177-2.104) | 3.67(-0.753-8.289) | 3.132(0.687-5.638) ^*^ | -0.021(-0.944-0.911) |
|  | 3 | 0.817(-0.299-1.945) | 3.856(-0.569-8.478) | 2.206(-0.177-4.646) | -0.053(-0.973-0.875) |
|  | 4 | 1.035(-0.077-2.16) | 5.484(1.012-10.154) ^*^ | 3.969(1.583-6.412) ^**^ | -0.045(-0.973-0.892) |
|  | 5 | 0.988(-0.123-2.112) | 3.749(-0.744-8.445) | 3.564(1.141-6.045) ^**^ | -0.071(-1.001-0.869) |
|  | 6 | 1.03(-0.09-2.162) | 6.329(1.717-11.151) ^**^ | 2.741(0.288-5.253) ^*^ | -0.05(-0.987-0.896) |
| Male | 0 | 0.934(-0.665-2.558) | -4.01(-12.936-5.831) | 0.599(-2.453-3.746) | -0.939(-2.57-0.72) |
|  | 1 | 1.666(0.169-3.185) ^*^ | 4.145(-1.619-10.247) | 3.126(-0.117-6.474) | -0.192(-1.497-1.131) |
|  | 2 | 0.627(-0.861-2.137) | 3.075(-2.647-9.133) | 3.077(-0.111-6.366) | 0.13(-1.085-1.359) |
|  | 3 | 0.754(-0.7-2.229) | 3.436(-2.274-9.479) | 1.687(-1.402-4.873) | -0.444(-1.649-0.776) |
|  | 4 | 1.051(-0.391-2.514) | 4.903(-0.861-11.002) | 3.269(0.183-6.45) ^*^ | 0.093(-1.13-1.33) |
|  | 5 | 0.913(-0.533-2.38) | 0.368(-5.318-6.396) | 2.188(-0.937-5.411) | 0.21(-1.017-1.452) |
|  | 6 | 1.299(-0.152-2.771) | 4.921(-0.996-11.191) | 2.734(-0.461-6.033) | -0.333(-1.564-0.914) |
| Female | 0 | 1(-0.881-2.916) | -0.614(-7.974-7.334) | 1.029(-2.607-4.801) | -0.078(-1.993-1.875) |
|  | 1 | 1.537(-0.235-3.34) | 3.108(-3.714-10.413) | 3.736(-0.096-7.715) | -1.052(-2.564-0.485) |
|  | 2 | 1.439(-0.307-3.215) | 4.612(-2.218-11.92) | 3.229(-0.542-7.143) | -0.21(-1.625-1.226) |
|  | 3 | 0.934(-0.798-2.696) | 4.459(-2.396-11.796) | 2.994(-0.712-6.838) | 0.491(-0.927-1.929) |
|  | 4 | 1.069(-0.67-2.838) | 6.44(-0.505-13.869) | 5.044(1.319-8.906) ^**^ | -0.215(-1.636-1.226) |
|  | 5 | 1.131(-0.599-2.892) | 8.797(1.639-16.46) ^*^ | 5.602(1.804-9.542) ^**^ | -0.435(-1.857-1.009) |
|  | 6 | 0.638(-1.113-2.419) | 8.476(1.266-16.199) ^*^ | 2.824(-0.96-6.753) | 0.353(-1.087-1.814) |
| Outpatient counts |  |  |  |  |  |
| RD in adults | 0 | 0.064(-0.126-0.255) | 2.655(2.032-3.282) ^**^ | 1.52(1.189-1.853) ^**^ | -0.653(-0.831--0.474) ^**^ |
|  | 1 | -0.106(-0.287-0.075) | 3.5(2.919-4.085) ^**^ | 1.319(0.98-1.659) ^**^ | 0.014(-0.133-0.16) |
|  | 2 | 0.479(0.303-0.655) ^**^ | 3.045(2.475-3.619) ^**^ | 1.72(1.376-2.065) ^**^ | 0.126(-0.015-0.267) |
|  | 3 | 0.563(0.393-0.734) ^**^ | 2.956(2.39-3.525) ^**^ | 1.97(1.633-2.308) ^**^ | 0.082(-0.056-0.221) |
|  | 4 | 0.671(0.5-0.842) ^**^ | 2.613(2.035-3.194) ^**^ | 1.876(1.541-2.213) ^**^ | 0.045(-0.092-0.182) |
|  | 5 | 0.588(0.415-0.761) ^**^ | 1.566(0.964-2.171) ^**^ | 2.099(1.762-2.438) ^**^ | -0.231(-0.366--0.097) ^**^ |
|  | 6 | 0.283(0.107-0.46) ^**^ | 1.925(1.345-2.508) ^**^ | 1.424(1.088-1.761) ^**^ | -0.117(-0.252-0.017) |
| RD in children | 0 | 0.499(0.245-0.754) ^**^ | 4.732(3.877-5.595) ^**^ | 3.676(3.226-4.128) ^**^ | -0.125(-0.568-0.245) |
|  | 1 | 0.458(0.212-0.704) ^**^ | 4.732(3.959-5.511) ^**^ | 3.552(3.087-4.019) ^**^ | 0.06(-0.135-0.256) |
|  | 2 | 0.756(0.51-1.003) ^**^ | 5.704(4.923-6.491) ^**^ | 4.042(3.573-4.514) ^**^ | 0.211(0.025-0.397) ^*^ |
|  | 3 | 1.208(0.965-1.452) ^**^ | 5.336(4.538-6.14) ^**^ | 3.657(3.198-4.118) ^**^ | 0.008(-0.175-0.192) |
|  | 4 | 1.465(1.221-1.709) ^**^ | 3.198(2.408-3.994) ^**^ | 3.473(3.01-3.938) ^**^ | -0.142(-0.528-0.527) |
|  | 5 | 1.054(0.807-1.301) ^**^ | 2.592(1.783-3.408) ^**^ | 3.493(3.028-3.959) ^**^ | -0.162(-0.846-0.529) |
|  | 6 | 0.714(0.467-0.96) ^**^ | 3.462(2.652-4.279) ^**^ | 3.24(2.783-3.7) ^**^ | -0.156(-0.836-0.320) |

^*^ *P* < 0.05, ^**^ *P* < 0.01 (Excess Risk is adjusted for temperature, relative humidity and atmospheric pressure, day of week, time trend and seasonality for mortality and hospital data, and public holiday only for hospital data).

**Supplementary Table S2** Excess Risk (95% confidence interval) of respiratory mortalities and outpatients per 10 μg/m^3^ increase of air pollutants for different lag days in ZS: single-pollutant model

| Variable | Lag | PM_2.5_ | SO_2_ | NO_2_ | O_3_ |
| --- | --- | --- | --- | --- | --- |
| Mortality counts |  |  |  |  |  |
| RD | 0 | 1.72(-0.386-3.871) | 0.698(-10.14-12.843) | 1.039(-2.315-4.509) | 0.882(-0.856-2.65) |
|  | 1 | 2.085(0.032-4.18)^*^ | 0.873(-9.451-12.376) | 0.763(-2.569-4.209) | 1.893(0.241-3.571) ^*^ |
|  | 2 | 1.572(-0.458-3.643) | 4.429(-5.685-15.628) | 0.259(-3.032-3.661) | 1.928(0.302-3.58) ^*^ |
|  | 3 | 1.188(-0.809-3.225) | 1.191(-8.658-12.101) | -0.822(-4.075-2.54) | 1.321(-0.31-2.978) |
|  | 4 | 0.414(-1.591-2.46) | -2.125(-11.966-8.817) | -0.171(-3.411-3.178) | -0.357(-1.971-1.284) |
|  | 5 | -0.299(-2.317-1.76) | -6.09(-15.726-4.648) | -2.579(-5.81-0.764) | -0.42(-2.021-1.206) |
|  | 6 | 1.461(-0.554-3.516) | -7.156(-16.759-3.554) | -0.606(-3.882-2.782) | 1.474(-0.153-3.128) |
| Male | 0 | 2.323(-0.67-5.407) | 0.263(-14.623-17.744) | 1.4(-3.436-6.478) | 1.296(-1.188-3.843) |
|  | 1 | 0.789(-2.192-3.861) | -3.967(-18.098-12.603) | 0.602(-4.185-5.629) | 0.149(-2.195-2.549) |
|  | 2 | 0.114(-2.84-3.157) | -3.249(-16.965-12.732) | -1.136(-5.866-3.832) | 1.516(-0.815-3.903) |
|  | 3 | 1.692(-1.202-4.672) | 3.823(-10.36-20.25) | 0.019(-4.708-4.98) | 2.442(0.102-4.836)* |
|  | 4 | 0.971(-1.922-3.95) | 3.295(-11.01-19.899) | 0.598(-4.135-5.565) | -1.003(-3.299-1.346) |
|  | 5 | 0.566(-2.354-3.573) | -1.136(-15.224-15.292) | -2.27(-7-2.7) | -0.286(-2.591-2.073) |
|  | 6 | 2.616(-0.29-5.607) | -1.67(-15.606-14.568) | 1.019(-3.766-6.042) | 1.79(-0.557-4.192) |
| Female | 0 | 1.153(-1.787-4.181) | 1.218(-13.853-18.925) | 0.782(-3.819-5.603) | 0.482(-1.932-2.955) |
|  | 1 | 3.226(0.418-6.112) ^*^ | 5.42(-8.974-22.09) | 1.026(-3.56-5.829) | 3.504(1.195-5.865) ^**^ |
|  | 2 | 2.866(0.091-5.718) ^*^ | 11.689(-2.583-28.053) | 1.558(-2.966-6.294) | 2.319(0.064-4.625) ^*^ |
|  | 3 | 0.737(-2.002-3.552) | -1.118(-14.288-14.075) | -1.509(-5.94-3.13) | 0.2(-2.061-2.512) |
|  | 4 | -0.086(-2.846-2.753) | -7.107(-20.101-8) | -0.792(-5.187-3.807) | 0.187(-2.07-2.497) |
|  | 5 | -1.087(-3.855-1.76) | -10.439(-23.109-4.319) | -2.805(-7.176-1.772) | -0.601(-2.815-1.663) |
|  | 6 | 0.424(-2.351-3.277) | -12.191(-24.878-2.639) | -2.011(-6.453-2.642) | 1.123(-1.123-3.42) |
| COPD | 0 | 1.308(-1.737-4.448) | -2.387(-17.644-15.697) | 3.547(-1.128-8.443) | -0.406(-2.781-2.027) |
|  | 1 | 0.332(-2.677-3.435) | -5.906(-20.28-11.059) | -0.806(-5.356-3.962) | 1.322(-0.964-3.661) |
|  | 2 | 1.855(-1.067-4.862) | 3.748(-10.687-20.515) | -0.043(-4.564-4.691) | 1.899(-0.366-4.217) |
|  | 3 | 1.6(-1.273-4.556) | 4.711(-9.683-21.4) | 1.449(-3.056-6.163) | 1.535(-0.724-3.845) |
|  | 4 | -0.153(-3.042-2.823) | -5.842(-19.668-10.364) | 0.812(-3.66-5.491) | -0.075(-2.318-2.219) |
|  | 5 | -0.363(-3.283-2.645) | -1.976(-16.198-14.658) | -1.82(-6.29-2.864) | -0.194(-2.419-2.082) |
|  | 6 | 2.377(-0.504-5.341) | -5.843(-19.764-10.494) | 0.125(-4.418-4.884) | 1.641(-0.612-3.945) |
| Male | 0 | 0.584(-3.632-4.984) | -12.198(-31.073-11.846) | 1.016(-5.486-7.965) | 0.141(-3.169-3.564) |
|  | 1 | -0.925(-5.149-3.486) | -8.878(-27.821-15.036) | -1.058(-7.437-5.761) | 0.584(-2.579-3.849) |
|  | 2 | 0.623(-3.48-4.901) | -0.206(-19.216-23.278) | -0.08(-6.462-6.736) | 1.134(-2.031-4.402) |
|  | 3 | 2.245(-1.804-6.461) | 15.035(-5.454-39.965) | 2.72(-3.688-9.555) | 3.138(-0.031-6.408) |
|  | 4 | -0.68(-4.756-3.57) | -1.046(-20.142-22.616) | -0.094(-6.436-6.678) | -1.018(-4.13-2.196) |
|  | 5 | -0.501(-4.64-3.818) | 2.731(-17.081-27.277) | -3.525(-9.854-3.247) | -0.298(-3.41-2.913) |
|  | 6 | 4.664(0.668-8.819) ^*^ | 5.461(-14.344-29.845) | 2.124(-4.348-9.034) | 1.133(-2.018-4.385) |
| Female | 0 | 2.051(-2.299-6.594) | 10.437(-12.9-40.027) | 5.996(-0.602-13.032) | -1.019(-4.397-2.479) |
|  | 1 | 1.603(-2.638-6.029) | -1.583(-22.154-24.424) | -0.503(-6.879-6.31) | 2.017(-1.257-5.399) |
|  | 2 | 3.133(-0.984-7.421) | 9.18(-11.628-34.887) | 0.03(-6.257-6.739) | 2.623(-0.594-5.945) |
|  | 3 | 0.978(-3.051-5.175) | -5.124(-24.178-18.719) | 0.238(-5.983-6.87) | -0.204(-3.399-3.097) |
|  | 4 | 0.378(-3.673-4.599) | -10.112(-28.957-13.734) | 1.64(-4.551-8.233) | 0.818(-2.388-4.129) |
|  | 5 | -0.214(-4.284-4.029) | -5.913(-25.173-18.305) | -0.181(-6.375-6.423) | -0.175(-3.334-3.087) |
|  | 6 | 0.029(-4.077-4.311) | -17.339(-35.525-5.977) | -1.707(-7.966-4.977) | 2.067(-1.13-5.367) |
| Outpatient counts |  |  |  |  |  |
| RD in adults | 0 | 0.079(-0.516-0.677) | 1.975(-0.788-4.816) | 2.139(1.039-3.251) ^**^ | 0.356(-0.124-0.838) |
|  | 1 | 0.284(-0.323-0.896) | 2.281(-0.537-5.178) | 1.164(0.084-2.256) ^*^ | 0.608(0.153-1.066) ^**^ |
|  | 2 | 0.484(-0.117-1.089) | 2.809(0.173-5.514) ^*^ | 1.078(0.009-2.157) ^*^ | 0.343(-0.116-0.804) |
|  | 3 | 0.623(0.019-1.23) ^*^ | 5.814(3.123-8.576) ^**^ | 1.911(0.871-2.962) ^**^ | 0.133(-0.35-0.618) |
|  | 4 | 0.327(-0.266-0.924) | 4.324(1.606-7.115) ^**^ | 2.489(1.437-3.552) ^**^ | 0.038(-0.424-0.502) |
|  | 5 | 0.83(0.23-1.433) | 4.883(2.078-7.766) ^**^ | 3.468(2.409-4.539) ^**^ | -0.05(-0.508-0.41) |
|  | 6 | 0.511(-0.097-1.123) | 4.638(1.897-7.453) ^**^ | 2.891(1.841-3.952) ^**^ | -0.178(-0.637-0.283) |
| RD in children | 0 | 0.776(0.056-1.502) ^*^ | 2.927(-0.422-6.388) | 6.919(5.534-8.321) ^**^ | 0.002(-0.595-0.603) |
|  | 1 | 0.67(-0.047-1.391) | 10.894(7.379-14.524) ^**^ | 6.659(5.307-8.027) ^**^ | 0.012(-0.546-0.572) |
|  | 2 | 0.869(0.139-1.603) ^*^ | 9.456(6.107-12.911) ^**^ | 5.474(4.158-6.807) ^**^ | 0.842(0.292-1.395) ^**^ |
|  | 3 | 1.201(0.463-1.944) ^**^ | 9.45(6.032-12.979) ^**^ | 5.744(4.432-7.072) ^**^ | -0.2(-0.772-0.375) |
|  | 4 | 1.779(1.052-2.512) ^**^ | 8.385(4.908-11.978) ^**^ | 8.018(6.672-9.381) ^**^ | -0.037(-0.597-0.526) |
|  | 5 | 0.843(0.106-1.584) ^*^ | 8.915(5.476-12.466) ^**^ | 7.541(6.189-8.911) ^**^ | 0.492(-0.061-1.047) |
|  | 6 | 0.17(-0.573-0.919) | 9.414(6.013-12.924) ^**^ | 5.646(4.328-6.981) ^**^ | 0.501(-0.057-1.062) |

^*^ *P* < 0.05, ^**^ *P* < 0.01 (Excess Risk is adjusted for temperature, relative humidity and atmospheric pressure, day of week, time trend and seasonality for mortality and hospital data, and public holiday only for hospital data); ^a^ Tertiary Hospital (RD); ^b^ Children Hospital (RD).

**Supplementary Table S3** The 95% confidence interval (CI) of the difference of ER between group variables

| Variable | Class | Pollutant | Diseases | Group | ER_1_ | ER_2_ | Stderr_1_ | Stderr_2_ | Lower limit of CI | Upper limit of CI |
| --- | --- | --- | --- | --- | --- | --- | --- | --- | --- | --- |
| HZ | Mortality | PM_2.5_ | COPD | Male: Female | 1.788 | 1.931 | 0.000781 | 0.000937 | -0.145391 | -0.140609 |
| HZ | Outpatient | SO_2_ | RD | Adults: children | 1.882 | 2.15 | 0.000336 | 0.000457 | -0.269112 | -0.266888 |
| HZ | Outpatient | NO_2_ | RD | Adults: children | 1.469 | 2.098 | 0.000189 | 0.000259 | -0.629628 | -0.628372 |
| ZS | Outpatient | SO_2_ | RD | Adults: children | 2.813 | 4.482 | 0.001377 | 0.001808 | -1.673454 | -1.664546 |
| ZS | Outpatient | NO_2_ | RD | Adults: children | 1.324 | 2.032 | 0.00067 | 0.000681 | -0.709872 | -0.706128 |
| Male | Mortality | PM_2.5_ | COPD | HZ: ZS | 1.788 | 4.795 | 0.000781 | 0.00213 | -3.011447 | -3.002553 |
| Adults | Outpatient | SO_2_ | RD | HZ: ZS | 1.882 | 2.813 | 0.000336 | 0.001377 | -0.933778 | -0.928222 |
| children | Outpatient | SO_2_ | RD | HZ: ZS | 2.15 | 4.482 | 0.000457 | 0.001808 | -2.335655 | -2.328345 |
| Adults | Outpatient | NO_2_ | RD | HZ: ZS | 1.469 | 1.324 | 0.000189 | 0.00067 | 0.1436356 | 0.1463644 |
| children | Outpatient | NO_2_ | RD | HZ: ZS | 2.098 | 2.032 | 0.000259 | 0.000681 | 0.064572 | 0.067428 |

**Supplementary Table S4 Excess Risk of respiratory mortalities and outpatients per 10 μg/m^3^ increase of air pollutants in both cities: multiple-pollutant model by season**

| Pollutant | Season | Class | Variable | ER(95% CI) in HZ ^a^ | ER(95% CI) in ZS ^a^ |
| --- | --- | --- | --- | --- | --- |
| PM_2.5_ | Cold | Mortality counts | RD | 1.489(0.420-2.570)^**^ | 1.793(-0.660-4.307) |
|  | Cold |  | COPD | 1.353(0.068-2.655)^*^ | 1.908(-1.568-5.507) |
|  | Warm |  | RD | -0.243(-2.743-2.323) | 3.614(-1.640-9.149) |
|  | Warm |  | COPD | 0.892(-2.228-4.111) | 5.629(-0.995-12.696) |
|  | Cold | Outpatient counts | Adults ^b^ | -0.038(-0.279-0.205) | 1.077(0.090-2.073)^*^ |
|  | Cold |  | Children ^c^ | 0.406(0.082-0.731)^*^ | 1.012(-0.027-2.007) |
|  | Warm |  | Adults ^b^ | -0.463(-0.833--0.091)^*^ | -1.305(-2.674-0.082) |
|  | Warm |  | Children ^c^ | -0.307(-0.81-0.199) | 3.209(1.356-5.096)^**^ |
| SO_2_ | Cold | Mortality counts | RD | 1.093(-3.248-5.630) | 1.403(-10.368-14.720) |
|  | Cold |  | COPD | 1.850(-3.660-7.675) | -1.399(-17.213-17.436) |
|  | Warm |  | RD | 2.209(-5.398-10.429) | 5.614(-24.494-47.726) |
|  | Warm |  | COPD | 2.817(-6.775-13.396) | -26.060(-53.760-18.233) |
|  | Cold | Outpatient counts | Adults ^b^ | 1.704(0.715-2.703)^**^ | 3.350(0.239-6.557)^*^ |
|  | Cold |  | Children ^c^ | 0.313(-1.012-1.656) | 1.687(-2.259-5.792) |
|  | Warm |  | Adults ^b^ | 1.845(0.667-3.036)^**^ | -8.493(-17.104-1.013) |
|  | Warm |  | Children ^c^ | 0.338(-1.186-1.885) | -0.461(-10.518-10.726) |
| NO_2_ | Cold | Mortality counts | RD | 3.209(0.812-5.664)^**^ | -2.297(-6.706-2.320) |
|  | Cold |  | COPD | 5.342(2.222-8.558)^**^ | -0.963(-7.602-6.153) |
|  | Warm |  | RD | -0.972(-4.788-2.997) | 0.125(-5.911-6.548) |
|  | Warm |  | COPD | -1.021(-5.812-4.014) | 13.568(5.485-22.270)^**^ |
|  | Cold | Outpatient counts | Adults ^b^ | 2.738(2.158-3.321)^**^ | -1.630(-3.643-0.425) |
|  | Cold |  | Children ^c^ | 1.940(1.118-2.769)^**^ | -0.270(-2.216-1.714) |
|  | Warm |  | Adults ^b^ | -0.007(-0.574-0.563) | 1.457(-0.792-3.758) |
|  | Warm |  | Children ^c^ | 0.123(-0.637-0.887) | -1.288(-3.764-1.251) |
| O_3_ | Cold | Mortality counts | RD | 0.661(-1.056-2.408) | 0.352(-2.413-3.195) |
|  | Cold |  | COPD | 1.106(-0.686-2.93) | 2.248(-2.108-6.798) |
|  | Warm |  | RD | -0.627(-2.092-0.861) | 3.368(1.262-5.517)^**^ |
|  | Warm |  | COPD | -1.262(-2.685-0.181) | 3.221(0.479-6.039)^*^ |
|  | Cold | Outpatient counts | Adults ^b^ | 1.301(0.917-1.687)^**^ | 1.228(0.262-2.204)^*^ |
|  | Cold |  | Children ^c^ | 0.119(-0.372-0.612) | 1.942(0.737-3.162)^**^ |
|  | Warm |  | Adults ^b^ | -0.182(-0.373-0.011) | 0.176(-0.450-0.807) |
|  | Warm |  | Children ^c^ | 0.075(-0.163-0.313) | -0.021(-0.772-0.737) |

^*^ *P* < 0.05, ^**^ *P* < 0.01 (Excess Risk is adjusted for temperature, relative humidity and atmospheric pressure, day of week, time trend and other pollutants for mortality and hospital data, and public holiday only for hospital data); ^a^ Excess Risk (95% confidence interval); ^b^ RD in adults; ^c^ RD in children.

**Supplementary Table S5**

Information on hospital and environmental monitoring station in HZ and ZS

| City | Variable | Name | Longitude (E) | Latitude (N) |
| --- | --- | --- | --- | --- |
| HZ | Tertiary Hospital | Hangzhou Red Cross Hospital | 120.194 | 30.270 |
| ZS | Tertiary Hospital | Zhoushan People's Hospital | 122.461 | 30.731 |
| HZ | Environmental monitoring station | Hemuxiaoxue | 120.131 | 30.316 |
| HZ | Environmental monitoring station | Xixi | 120.062 | 30.275 |
| HZ | Environmental monitoring station | Yunxi | 120.088 | 30.182 |
| HZ | Environmental monitoring station | Zhejiangnongda | 120.190 | 30.269 |
| HZ | Environmental monitoring station | Binjiang | 120.211 | 30.210 |
| HZ | Environmental monitoring station | Xiasha | 120.348 | 30.306 |
| HZ | Environmental monitoring station | Wolongqiao | 120.127 | 30.246 |
| HZ | Environmental monitoring station | Zhaohuiwuqu | 120.157 | 30.290 |
| ZS | Environmental monitoring station | Linchengxinqu | 122.190 | 29.994 |
